# Prioritization framework for improving the value of care for very low birth weight and very preterm infants

**DOI:** 10.1038/s41372-021-01114-6

**Published:** 2021-06-01

**Authors:** Brian C. King, Troy Richardson, Ravi M. Patel, Henry C. Lee, Nicolas A. Bamat, Matthew Hall, Jonathan L. Slaughter

**Affiliations:** 1 Department of Pediatrics, Section of Neonatology, Baylor College of Medicine and Texas Children’s Hospital, Houston, TX USA; 2 Children’s Hospital Association, Lenexa, KS USA; 3 Division of Neonatology, Emory University School of Medicine and Children’s Healthcare of Atlanta, Atlanta, GA USA; 4 Division of Neonatology, Stanford University, Stanford, CA USA; 5 Division of Neonatology, Children’s Hospital of Philadelphia, Perelman School of Medicine at the University of Pennsylvania, Philadelphia, PA USA; 6 Division of Neonatology, Nationwide Children’s Hospital, The Ohio State University, Columbus, OH USA

**Keywords:** Health care economics, Paediatrics, Health services

## Abstract

**Objective:**

Create a prioritization framework for value-based improvement in neonatal care.

**Study design:**

A retrospective cohort study of very low birth weight (<1500 g) and/or very preterm (<32 weeks) infants discharged between 2012 and 2019 using the Pediatric Health Information System Database. Resource use was compared across hospitals and adjusted for patient-level differences. A prioritization score was created combining cost, patient exposure, and inter-hospital variability to rank resource categories.

**Results:**

Resource categories with the greatest cost, patient exposure, and inter-hospital variability were parenteral nutrition, hematology (lab testing), and anticoagulation (for central venous access and therapy), respectively. Based on our prioritization score, parenteral nutrition was identified as the highest priority overall.

**Conclusions:**

We report the development of a prioritization score for potential value-based improvement in neonatal care. Our findings suggest that parenteral nutrition, central venous access, and high-volume laboratory and imaging modalities should be priorities for future comparative effectiveness and quality improvement efforts.

## Introduction

As national healthcare expenditures have grown, increasing attention has been paid to value (i.e., quality of health outcomes per dollar spent) across medical fields, including neonatology [1, 2]. Identifying high-yield opportunities to reduce waste and improve value can be challenging. Past efforts to set priorities for neonatal care have relied on expert opinion. For example, in 2015 the Choosing Wisely Campaign, an initiative to advance a national dialogue on avoiding unnecessary medical tests, treatments, and procedures, published their top five list in newborn care based on expert consensus without objective cost and utilization data [3]. More objective methods of priority setting are needed to focus value improvement efforts on high yield targets. Recent work has estimated the cost of individual test and treatment categories used in neonatal care, however, cost estimates alone are insufficient for prioritization [4].

Unwarranted practice variation has been identified as a key contributor to health care inefficiency and waste [5, 6]. Prior work has established significant inter-hospital variation beyond what is expected from differences in the patient population for many neonatal practices [7–11]. In some instances, unwarranted practice variation has been linked to variation in patient outcomes [12–14]. Much of this variation likely reflects the uncertainty of evidence and provider-specific preferences [15]. These highly variable care patterns represent opportunities for reducing unnecessary and wasteful care by establishing “best practices” for use either through comparative effectiveness research or quality improvement efforts [16]. Comparative effectiveness research is clinical and epidemiological research focused on determining what health interventions, or a combination of health interventions, achieve the best outcomes [17]. Practice variation can be a useful tool in priority setting as greater variation suggests a greater opportunity for change and optimization. While practice variation has been used more broadly in pediatrics to prioritize research [18, 19], it has not been used in efforts to develop a priority-setting framework for neonatal care.

Therefore, our objectives were to estimate the inter-hospital variability of clinician-driven tests and treatments (CTTs) among very low birth weight and very preterm infants during their birth hospitalization, and create an objective prioritization framework for value-based improvements in neonatal care by combining data on cost, use and inter-hospital practice variation.

## Methods

### Study design

We conducted a retrospective cohort study of very low birth weight (VLBW, birth weight <1500 g) and very preterm (VP, gestational age (GA) <32 weeks) infants admitted to neonatal intensive care units (NICUs) in the United States (US) children’s hospitals affiliated with the pediatric health information system (PHIS) database and discharged from 2012 to 2019, to estimate the cost and inter-hospital variability of CTTs ordered during hospitalization.

### Data source

PHIS is an administrative database containing hospitalization data from 51 tertiary-care children’s hospitals, maintained by the Children’s Hospital Association (Lenexa, KS). The database contains data on demographics, diagnosis and procedure codes (using *International Classification of Diseases, Ninth and Tenth Revision, Clinical Modification* [ICD-9, ICD-10]), and daily resource utilization. Resources at each hospital are mapped to a common set of clinical transaction codes which are organized into imaging studies, clinical services, laboratory tests, pharmacy, supplies, and other (e.g., room) charges. IBM Watson Health (Ann Arbor, MI) manages the data warehouse function for the database. Data are subjected to reliability and validity checks and must pass a specified threshold of quality before being incorporated into the database. All personal health information is deidentified within the database. A protocol was reviewed by the Baylor College of Medicine Institutional Review Board and was not considered human subjects research.

### Cohort identification

Subjects were identified as either VLBW or VP by discrete data for GA and birth weight (BW), or by diagnostic code if discrete data were unavailable. Subjects less than 22 weeks’ gestation or <400 g were excluded. Subjects admitted after 1 day of age were excluded since we were unable to measure resource use at the referring hospitals. Utilization and cost were only considered for days with a NICU bed charge to exclude costs incurred in other units (e.g., pediatric intensive care units). We excluded subjects with congenital anomalies that could significantly impact care costs using ICD-9/10 diagnosis codes. Two authors (BCK, JLS) reviewed all ICD-9/10 diagnosis codes among the potential cohort and independently assigned codes for exclusion. Disagreements were settled by consensus. Excluded diagnoses included critical congenital heart defects and congenital malformations of other organ systems (renal, lung, etc.). Diagnosis codes for a patent ductus arteriosus and atrial septal defects, common diagnoses among preterm infants, were not excluded. To account for potential data entry errors, we utilized a number of other exclusions which have been previously described [20]. In addition, we also excluded any hospital with fewer than 100 patients meeting our inclusion and exclusion criteria during the study period to ensure each included hospital had a sample size large enough to accurately estimate median utilization rates for between-hospital comparisons.

### Outcomes reported

Pharmaceutical, laboratory, and imaging billing were classified into clinically relevant CTT categories (e.g., chest radiographs, antibiotics) and costs were estimated, as previously described [20]. In brief, costs are estimated from hospital charges, regionally adjusted using the CMS wage/price index and adjusted to 2019 US dollars using the producer price index for inpatient services, which is considered the best available tool for inflation of inpatient hospital costs [21]. To account for variation in billed charges across PHIS hospitals, standardized costs were applied to all encounters using the median cost for each billing item across all hospitals [18]. Exposure to a CTT category was defined as at least one charge for any component of that CTT on at least one day during their NICU stay. We defined NICU hospitalizations that were in the top ten percent of CTT-related costs as Resource Intensive NICU stays. Physician billing and procedure costs are not available within the PHIS database and were therefore not included in the analysis. The costs of nutritional fortification for enteral feeding were also not estimated because they are not routinely billed separately from the daily room and board charges.

### Severity of illness

We assessed patient-level severity of illness using a length-of-stay-based relative weight approach. We adapted the Hospitalization Resource Intensity Score for iKds (H-RISK) by restricting the all-patient refined diagnosis-related groups (APR-DRGs; 3M Health Information Systems, Salt Lake City, UT) to only those admitted to PHIS NICUs and by basing our calculation of relative weights on NICU days [22] These relative weights were calculated as the ratio of mean NICU LOS for each APR-DRG relative to the overall mean LOS among all infants admitted to NICUs included in PHIS. Mean values were Winsorized (i.e., extreme high and low outlying values were replaced with the 95th and 5th percentiles respectively) and NICU mortalities were excluded to minimize the influence of extreme outliers on relative weight calculations. Relative weights (the NICU-SOI score) were then applied to our cohort based on the APR-DRG assigned to each NICU stay. A NICU-SOI score is greater than 1 means that their NICU stay was assigned an APR-DRG with a mean NICU LOS that is longer than the average NICU encounter for included NICUs. Increasing values of the NICU-SOI score indicate increasing mean NICU LOS for the assigned APR-DRG, suggestive of higher severity of illness.

### Statistical analysis

We summarized categorical variables using frequencies and percentages, non-normally distributed continuous variables using medians and interquartile range (IQR), and normally distributed variables using mean and standard error (SE). Demographics in PHIS include sex, GA, birth weight, age at admission, admission source, race/ethnicity, insurance type, median household income, and disposition at hospital discharge (including mortality). We compared demographics and the NICU-SOI score between resource-intense NICU stays and resource mild/moderate NICU stays using a chi-square test for the association for categorical variables, a Wilcoxon rank-sum test for non-normally distributed continuous variables, and a two-sample *t*-test for normally-distributed continuous variables. Multivariable generalized linear mixed models (GLMM) were used to identify demographic and clinical characteristics associated with overall CTT-related spending and the odds of having a resource-intensive NICU day. Covariates in the model were the demographics listed above and our calculated NICU-SOI score. All GLMMs included a random hospital effect to account for the clustering of NICU patients at the same hospital.

We assessed hospital-to-hospital variation in total CTT-related costs by estimating the intraclass correlation coefficient (ICC) as the percentage of total variation attributable to hospital variation after adjusting for patient demographics and our NICU-SOI. Race/ethnicity was included as a variable because of its association with quality of care[23]. We identified high and low outlying hospitals for total adjusted per-patient CTT-related costs and specific billing group (pharmaceutical, laboratory, imaging) costs by comparing hospital medians to the cohort overall median and IQR. High- and low-cost outlier hospitals were defined as hospitals with a median adjusted per-patient CTT-related cost greater or less than the population third and first quartile, respectively.

Next, we created a variability index that estimates hospital-to-hospital variation in utilization and allows for direct comparisons across CTT categories. The variability index was derived to capture two types of inter-hospital variation; variation in the proportion of patients exposed to a given CTT category at least once (“exposure variability”), and variation in utilization of the CTT category among those exposed (“utilization variability”). Exposure variability was calculated using the adjusted percent exposure in the entire cohort (i.e., mean exposure) and the measured spread of adjusted hospital-specific exposures around the mean using standard distances. Exposure variability was estimated by calculating the standard deviation of those standard distances. This approach estimates variability in the proportion of infants exposed for each CTT category at each hospital beyond what would be expected to occur by chance when sampling from the overall population. Utilization variability was calculated using CTT-related costs, which act as a surrogate for utilization in this case because the PHIS database applies an average cost estimate to all patients across included hospitals. Higher costs on a given hospital day (and/or a greater number of hospital days with related costs) mean more utilization of that CTT category. Utilization Variability was estimated by calculating the coefficient of variation (CV) of the adjusted hospital mean total costs among exposed patients. The variability index for a CTT category was then calculated as the standardized Euclidean distance of the exposure and utilization variabilities described above. To minimize the impact of the hospital to hospital variation in exposure due to differences in billing patterns (rather than variation in physician and hospital practice patterns), we excluded hospitals from an individual CTT category variability index calculation if their hospital exposure rate was more than four times or less than one-quarter of the overall population exposure rate for a given CTT category.

Lastly, we developed an overall prioritization score for each CTT category by calculating the standardized Euclidean distance (from the origin) based on three factors; total adjusted costs, the proportion of patients exposed, and variability. A flowsheet of the methodology to create the prioritization score is included in the online supplement (Supplemental Fig. 2). All components were standardized using standard deviations to mitigate the influence of any one component on the overall distance calculation. Larger prioritization score values indicate greater costs, higher volumes, or higher hospital-to-hospital variation (weighted equally). *P*-values less than 0.05 were considered statistically significant. All data management and analyses were conducted using SAS v9.4 (SAS Institute, Cary, NC).

## Results

### Cohort demographics and risk of resource-intensive NICU stay

We identified 26,098 subjects across 40 children’s hospitals contributing 1,373,883 total NICU days which met our inclusion and exclusion criteria. Ten hospitals were excluded for low patient volume, and one was excluded because it did not provide consistent data during the study period, with a gap in annual neonatal admissions recorded. A flow diagram with our inclusion and exclusion criteria is included (Supplemental Fig. 1). Patient demographics for the entire cohort are summarized in Table 1. On multivariable logistic regression analysis, decreasing GA, male sex, Black race, outborn admission, and a higher NICU-SOI score were all associated with significantly higher odds of a resource-intensive NICU stay, while mortality and self-pay insurance (compared to commercial insurance) were associated with lower odds of a resource-intensive NICU stay (Table 1). On sensitivity analysis, when our NICU-SOI score was excluded, the odds of a resource-intensive NICU stay increased as the birth weight category decreased (Supplemental Table 1). A secondary analysis using linear regression and total CTT-related costs showed similar results (Supplemental Table 2).

**Table 1:** Cohort demographics and adjusted odds ratio of a resource-intensive NICU stay.

Patient demographics	Total	CTT resource mild/moderate NICU stay	CTT resource-intense NICU stay^a^	Adjusted odds ratio (95% CI)^b^
Total	26,098	23,488	2610	
Sex, *N* (%)				
Male	13,462 (51.6)	12,063 (51.4)	1399 (53.6)	1.22 (1.09,1.36)
Female	12,613 (48.3)	11,402 (48.5)	1211 (46.4)	REF
Gestational age (weeks), *N* (%)^c^				
22	158 (0.6)	120 (0.5)	38 (1.5)	4.77 (2.33,9.77)
23–24	2762 (10.6)	1813 (7.7)	949 (36.4)	8.97 (5.09,15.81)
25–26	3991 (15.3)	3134 (13.3)	857 (32.8)	6.14 (3.53,10.70)
27–28	5282 (20.2)	4891 (20.8)	391 (15.0)	2.86 (1.67,4.92)
29–30	6946 (26.6)	6717 (28.6)	229 (8.8)	2.07 (1.22,3.52)
31	4665 (17.9)	4595 (19.6)	70 (2.7)	1.75 (0.97,3.15)
>31 weeks	1676 (6.4)	1658 (7.1)	18 (0.7)	REF
Not available	618 (2.4)	560 (2.4)	58 (2.2)	4.22 (2.23,7.98)
Birth weight (g), *N* (%)^c^				
400–499	344 (1.3)	224 (1.0)	120 (4.6)	1.65 (1.00,2.73)
500–749	3902 (15.0)	2715 (11.6)	1187 (45.5)	0.99 (0.66,1.50)
750–999	4862 (18.6)	4133 (17.6)	729 (27.9)	0.91 (0.62,1.34)
1000–1249	5465 (20.9)	5150 (21.9)	315 (12.1)	0.80 (0.56,1.15)
1250–1499	6447 (24.7)	6258 (26.6)	189 (7.2)	1.04 (0.74,1.47)
>1499 g	4916 (18.8)	4848 (20.6)	68 (2.6)	REF
Not available	162 (0.6)	160 (0.7)	2 (0.1)	0.84 (0.15,4.57)
Race/ethnicity, *N* (%)				
Non-Hispanic White	11,170 (42.8)	10,077 (42.9)	1093 (41.9)	REF
Non-Hispanic Black	6133 (23.5)	5305 (22.6)	828 (31.7)	0.84 (0.73,0.97)
Hispanic	4151 (15.9)	3797 (16.2)	354 (13.6)	0.93 (0.76,1.13)
Asian	863 (3.3)	816 (3.5)	47 (1.8)	0.63 (0.44,0.91)
Other	3781 (14.5)	3493 (14.9)	288 (11.0)	0.75 (0.62,0.90)
Admission source, *N* (%)				
Inborn	7604 (29.1)	7040 (30.0)	564 (21.6)	REF
Outborn	16,463 (63.1)	14,788 (63.0)	1675 (64.2)	2.12 (1.72,2.61)
Other	2031 (7.8)	1660 (7.1)	371 (14.2)	2.27 (1.43,3.59)
Age at Admission (days), *N* (%)				
0	24,883 (95.3)	22,452 (95.6)	2431 (93.1)	REF
1	1215 (4.7)	1036 (4.4)	179 (6.9)	0.96 (0.77,1.21)
Insurance Type, *N* (%)				
Commercial	9690 (37.1)	8859 (37.7)	831 (31.8)	REF
Government	15,634 (59.9)	13,904 (59.2)	1730 (66.3)	0.95 (0.84,1.08)
Self-pay	289 (1.1)	273 (1.2)	16 (0.6)	0.38 (0.20,0.71)
Other	485 (1.9)	452 (1.9)	33 (1.3)	0.70 (0.44,1.11)
Disposition at discharge, *N* (%)				
Home	18,886 (72.4)	17,047 (72.6)	1839 (70.5)	REF
Transfer	4646 (17.8)	4172 (17.8)	474 (18.2)	1.23 (1.05,1.45)
Mortality	2340 (9.0)	2063 (8.8)	277 (10.6)	0.13 (0.11,0.16)
Other	226 (0.9)	206 (0.9)	20 (0.8)	6.84 (3.86,12.13)
The median household income quartile				
Q1	7479 (28.7)	6544 (27.9)	935 (35.8)	1.14 (0.95,1.36)
Q2	6829 (26.2)	6137 (26.1)	692 (26.5)	0.96 (0.80,1.15)
Q3	5973 (22.9)	5429 (23.1)	544 (20.8)	0.98 (0.82,1.17)
Q4	5228 (20.0)	4826 (20.5)	402 (15.4)	REF
NICU-SOI Score, Mean (SE)^d^	3.21 (0.011)	2.96 (0.01)	5.48 (0.03)	2.03 (2.01,2.05)

^a^Top the tenth percentile of CTT-related costs during NICU birth hospitalization.

^b^Adjusted Odds Ratio for resource-intensive NICU stays, using multiple logistic regression.

^c^Reported as ordinal categories due to a combination of continuous GA/BW data entry and ICD-9/10 diagnostic code definitions.

^d^NICU-SOI Score is a LOS-based relative weight based on the patient’s assigned APR-DRG SOI score and the average NICU length of stay among included children’s hospitals.

### Inter-hospital variation in total and billing group adjusted CTT-related costs

**Fig. 1:**
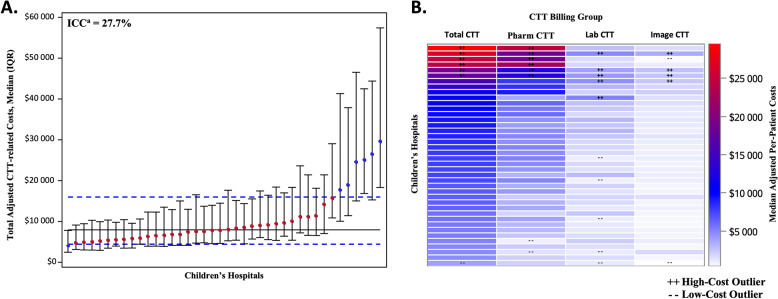
Variability in median (IQR) adjusted per-patient total CTT-related costs (A) and billing group heat map (B) across included US Children’s Hospitals. Hospital outliers were defined as hospitals with median adjusted costs greater/less than the inter-quartile range of the entire population. Within the billing group heat map (**B**), rows represent individual children’s hospitals, and columns are median adjusted per-patient cost estimates for each billing group and total CTT-related costs. ^a^Intraclass correlation coefficient estimates the amount of total variation due to inter-hospital variation after accounting for patient demographics and illness severity.

Inter-hospital variation in total and billing group adjusted CTT-related costs are reported in Fig. 1. The ICC, an estimate of the percentage of variation explained by inter-hospital variation (as opposed to patient-level severity of illness, defined by our model to include demographic variables and the LOS-based case mix index), was 27.7% for total CTT-related costs. Six hospitals (15%) were outliers in total adjusted CTT-related costs, defined as having a median cost greater than the upper quartile range for the overall population. The median per-patient adjusted CTT-related costs at those six hospitals were all more than twice the overall population median ($17,801 for the lowest of the high-cost outliers, compared to $7942 for the population median). Among the three billing groups, pharmaceutical adjusted CTT-related costs had the widest variation across hospitals. The six high-cost outlier hospitals among pharmaceutical adjusted CTT-related costs were the same six high-cost outliers in overall spending. Laboratory and Imaging adjusted CTT-related costs had five and four outlier hospitals, respectively, but the overall differences in spending among those billing groups were smaller than that for pharmaceutical spending (Fig. 1b).

### Prioritization of CTTs

**Fig. 2:**
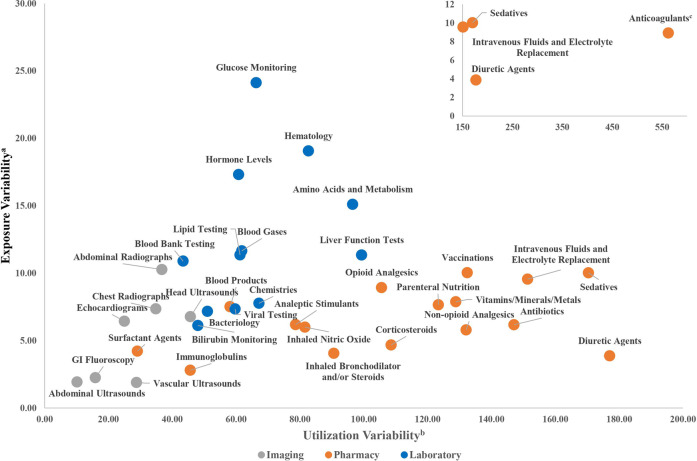
Scatter plot of exposure and utilization variability for CTT categories. ^a^Exposure variability is the standard deviation of the standard distances of adjusted hospital exposure proportions from the mean population exposure proportion for each CTT category. ^b^Utilization variability is the coefficient of variation of the adjusted hospital mean costs per exposed patients. ^c^Utilization variability for anticoagulants exceeded the x-axis limit of the larger figure. Three other CTT categories are repeated in inset for reference.

Adjusted total cost and descriptive measures of CTT variability are reported in Table 2, ranked by their prioritization score. Parenteral nutrition, chemistries, and anticoagulants were the costliest CTT categories, responsible for a combined total cost of $111,373,888 (40% of the cumulative cost of all CTT categories included). Chest radiographs were the costliest Imaging CTT ($14,852,629) but ranked fifth in total cost across all billing groups. Exposure Variability and Utilization Variability for each CTT category are shown on a scatter plot (Fig. 2). Based on our calculated inter-hospital variability index which combines those two variability estimates, anticoagulants, glucose monitoring, and hematology laboratory tests were the most variable overall. The imaging CTT with the highest inter-hospital variability was abdominal radiographs, but they were ranked eighth overall based on the inter-hospital variability index.

**Table 2:** Prioritization of clinician-driven tests and treatments.

			Ranks		Exposed	Cost	Inter-hospital variability			
CTT category	Billing group	Prioritization score^a^	Cost	Variability	*N* (%)	Adjusted cost (cumulative % total costs)^b^	Median patient exposure, % (IQR)	Median NICU days, % (IQR)	Hospital median cost per exposed patient, $ (IQR)	Variability index^c^
Parenteral Nutrition	Pharmaceutical	6.56	1	14	23,752 (89%)	$58,411,076 (21%)	95% [89%,97%]	28% [24%,37%]	$2454 [$1127,$3178]	3.21
Anticoagulants	Pharmaceutical	6.00	3	1	19,940 (75%)	$26,047 954 (30%)	84% [72%,92%]	12.5% [8%,19%]	$63 [$20,$129]	5.83
Hematology	Laboratory	5.04	12	3	25,092 (95%)	$8,187,120 (33%)	99% [96%,100%]	26% [18%,34%]	$299 [$214,$493]	4.66
Glucose Monitoring	Laboratory	4.85	15	2	22,676 (85%)	$5,616,816 (35%)	98% [85%,100%]	28% [14%,39%]	$237 [$125,$397]	4.67
Chemistries	Laboratory	4.65	2	17	25,086 (95%)	$26,914 858 (45%)	98% [96%,99%]	31% [26%,44%]	$1011 [$728,$1519]	3.05
Intravenous Fluids and Electrolyte Replacement	Pharmaceutical	4.55	8	6	24,032 (91%)	$9,719,875 (48%)	96% [87%,99%]	30.4% [24%,41%]	$250 [$150,$517]	4.00
Blood gases	Laboratory	4.46	4	15	22,425 (84%)	$25,385 871 (57%)	93% [85%,97%]	28% [18%,38%]	$1154 [$712,$1557]	3.20
Amino acids and Metabolism	Laboratory	4.25	24	4	13,956 (53%)	$2,048,566 (58%)	66% [32%,87%]	4% [1%,10%]	$104 [$76,$146]	4.59
Head Ultrasounds	Imaging	4.07	11	11	21,588 (81%)	$8,635,416 (61%)	87% [78%,91%]	4% [4%,6%]	$370 [$325,$456]	3.57
Chest Radiographs	Imaging	4.04	5	16	22,930 (86%)	$14,852 629 (66%)	92% [86%,96%]	14% [10%,19%]	$608 [$506,$797]	3.09
Vitamins/Minerals/Metals	Pharmaceutical	4.01	20	12	23,583 (89%)	$3,757,705 (67%)	92% [85%,96%]	61% [43%,67%]	$96 [$33,$230]	3.31
Liver Function Tests	Laboratory	3.92	25	5	13,408 (51%)	$1,665,320 (68%)	59% [29%,76%]	4% [1%,8%]	$58 [$42,$145]	4.20
Vaccinations	Pharmaceutical	3.76	23	7	14,824 (56%)	$2,110,849 (69%)	58% [43%,68%]	2% [1%,2%]	$168 [$83,$252]	3.88
Abdominal Radiographs	Imaging	3.76	13	8	15,528 (59%)	$6,097,284 (71%)	65% [54%,80%]	7% [3%,9%]	$358 [$267,$439]	3.77
Antibiotics	Pharmaceutical	3.55	16	20	21,848 (82%)	$5,181,476 (73%)	86% [80%,93%]	17% [12%,22%]	$147 [$101,$261]	2.75
Bilirubin Monitoring	Laboratory	3.51	18	28	24,463 (92%)	$4,546,251 (74%)	96% [93%,97%]	17% [13%,21%]	$147 [$130,$237]	2.22
Hormone levels	Laboratory	3.51	31	10	14,574 (55%)	$982,116 (75%)	59% [28%,91%]	2% [1%,3%]	$77 [$40,$113]	3.63
Bacteriology	Laboratory	3.50	17	26	23,362 (88%)	$4,617,621 (76%)	91% [85%,96%]	6% [5%,11%]	$174 [$127,$210]	2.41
Analeptic Stimulants	Pharmaceutical	3.41	7	24	20,701 (78%)	$10,046 125 (80%)	81% [75%,86%]	54% [49%,62%]	$456 [$260,$714]	2.54
Opioid Analgesics	Pharmaceutical	3.37	29	9	10,648 (40%)	$1,079,652 (80%)	45% [32%,55%]	7% [4%,11%]	$69 [$26,$95]	3.65
Blood bank testing	Laboratory	3.32	22	23	20,811 (78%)	$2,384,766 (81%)	86% [79%,92%]	2% [2%,3%]	$103 [$85,$150]	2.55
Lipid testing	Laboratory	3.15	26	19	17,392 (66%)	$1,343,400 (82%)	82% [70%,88%]	7% [4%,10%]	$59 [$46,$103]	2.81
Sedatives	Pharmaceutical	2.88	36	13	6282 (24%)	$468,759 (82%)	23% [15%,35%]	4% [2%,9%]	$40 [$17,$84]	3.29
Echocardiograms	Imaging	2.73	6	25	10,273 (39%)	$13,971 996 (87%)	41% [33%,50%]	3% [2%,3%]	$1363 [$1 189,$1482]	2.45
Blood Products	Pharmaceutical	2.69	19	18	8541 (32%)	$3,916,533 (88%)	36% [30%,48%]	3% [1%,6%]	$498 [$254,$629]	2.89
Viral testing	Laboratory	2.47	27	21	6970 (26%)	$1,222,943 (89%)	24% [19%,34%]	1% [1%,2%]	$145 [$117,$221]	2.71
Non-opioid Analgesics	Pharmaceutical	2.41	21	22	8265 (31%)	$3,203,314 (90%)	30% [25%,36%]	2% [2%,4%]	$309 [$133,$531]	2.55
Surfactant Agents	Pharmaceutical	2.10	9	33	10,875 (41%)	$8,794,328 (93%)	43% [38%,48%]	1% [1%,2%]	$856 [$667,$996]	1.69
Immunoglobulins	Pharmaceutical	2.07	10	35	4497 (17%)	$8,674,122 (96%)	5% [4%,7%]	1% [0%,1%]	$5526 [$4 257,$7 977]	1.18
Diuretic agents	Pharmaceutical	2.05	34	29	7553 (28%)	$752,242 (96%)	28% [25%,34%]	13% [9%,19%]	$54 [$34,$95]	2.15
Corticosteroids	Pharmaceutical	1.87	30	30	5050 (19%)	$1,055,401 (97%)	20% [16%,23%]	5% [4%,9%]	$110 [$47,$211]	2.07
Vascular Ultrasounds	Imaging	1.70	35	31	1694 (6%)	$708,383 (97%)	6% [6%,8%]	0.2% [0.1%,0.3%]	$375 [$335,$396]	1.97
Inhaled Bronchodilators and/or Steroids	Pharmaceutical	1.56	33	32	2667 (10%)	$776,060 (97%)	9% [6%,13%]	3% [1%,7%]	$136 [$65,$373]	1.78
Inhaled Nitric Oxide	Pharmaceutical	1.28	14	27	1845 (7%)	$5,854,650 (99%)	17% [9%,24%]	0.3% [0.2%,0.5%]	$1002 [$565,$1375]	2.26
GI Fluoroscopy	Imaging	1.10	32	34	2674 (10%)	$814,412 (99%)	10% [8%,12%]	0.3% [0.2%,0.4%]	$294 [$277,$321]	1.23
Abdominal Ultrasounds	Imaging	0.89	28	36	4071 (15%)	$1,192,815 (100%)	16% [14%,17%]	0.5% [0.4%,0.7%]	$291 [$273,$312]	0.86

^a^Prioritization score is the standardized Euclidean distance for the cost and variability index, adjusted by the number of subjects exposed.

^b^Cumulative percentage of included CTT-related costs.

^b^Variability index is the standardized Euclidean distance of exposure variability and utilization variability.

**Fig. 3:**
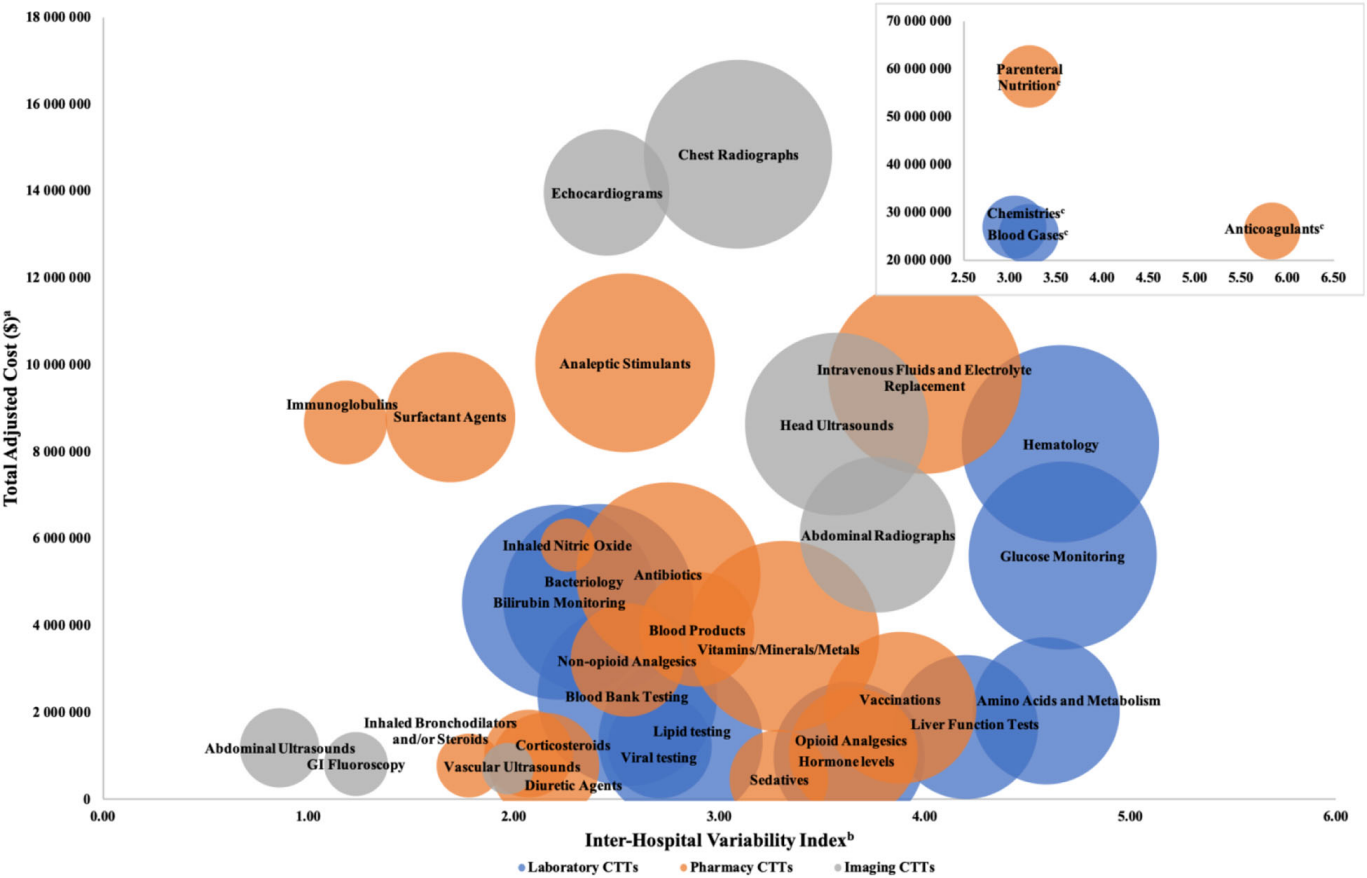
Prioritization framework for value-driven comparative effectiveness research and quality improvement. Size of the bubbles represents the percentage of the total cohort exposed to each CTT-category. ^a^Adjusted for patient demographics (sex, gestational age, birth weight, age at admission, admission source, race/ethnicity, insurance type, median household income, and disposition at hospital discharge) and our NICU-SOI score. ^b^Inter-Hospital Variability Index is the standardized Euclidian distance of Exposure and Utilization Variability. ^c^Parenteral nutrition, Anticoagulants, Chemistries and Blood gases exceeded the y-axis limit for the larger figure.

The components of our prioritization score (total cost, variability, population exposure) are represented by a bubble chart that plots the inter-hospital variability index by the total cost (Fig. 3). The top 3 CTT categories with the highest prioritization scores were parenteral nutrition, anticoagulants, and hematology, which together were responsible for 33% of the cumulative cost of all included CTT categories. Of the top 10 CTT categories for prioritization, three are pharmaceuticals, five are laboratory testing categories, and two are imaging tests and combined accounted for 66% of the cumulative cost of all included CTT categories.

## Discussion

We report the first value-based prioritization framework for comparative effectiveness research and quality improvement initiatives in the care of preterm infants. Combining estimates of cost, exposure, and inter-hospital variability into a single prioritization score, we ranked test and treatment categories to identify targets for further research with the highest potential for improving the value of neonatal care. Among a cohort of 26,098 infants across 8 years, the top 10 high priority CTT categories were responsible for $185,820,182 in costs (66% of all costs from included CTT categories), and include many commonly used tests and treatments in neonatal care, suggesting value-based improvements should focus on optimizing our approach to routine neonatal care. The use of parenteral nutrition was identified as the highest priority overall, followed by anticoagulants (including use for central line patency), and a number of commonly used laboratory test categories (hematology, glucose monitoring, chemistries, blood gases).

Estimates of variability have been used previously to prioritize value-driven efforts. Lee et al. measured cost variability to prioritize a value-driven outcomes program and found prematurity had the third-highest variation indirect costs among inpatient and outpatient diagnoses, highlighting the importance of focusing on variability in preterm infant care [24]. Keren et al. and Cameron et al. used variation in total cost to establish priorities for comparative effectiveness research among inpatient pediatric diagnoses and pediatric surgical diagnoses, respectively [18, 19]. These studies were not designed to identify specific drivers of cost variation within each population. Our study similarly uses a value-based priority-setting approach but is novel in identifying the key drivers of resource-related cost and variability within a specific neonatal population. This is in line with work done by Providence St. Joseph Health system, which has used detailed data on practice variation to drive their value-oriented architecture program to improve outcomes and reduce costs within specific clinically relevant patient populations [25].

Practices identified by our prioritization score may represent potential opportunities for targeted deimplementation of routine tests and treatments. Deimplementation science is the process of identifying low-value services that can be safely eliminated or reduced in practice [26, 27]. While deimplementation may involve broadly eliminating wasteful practices, our high priority targets require a more nuanced approach. For example, parenteral nutrition had the highest prioritization score overall, due to its high cost, frequent use, and wide variability. The optimal use of parenteral nutrition and feeding practices in preterm populations are areas of uncertainty and active study [28–30]. Prioritizing parenteral nutrition as a target for value-based practice improvement should focus on identifying specific opportunities for reduction, such as older stable preterm infants who may safely tolerate faster feeding advancement, rather than broad elimination strategies. A focus on targeted faster feeding advancement would also address anticoagulation, another category that ranked highly on our prioritization score, as it would have the potential to reduce central line days. Similar efforts would be needed for other high-priority CTT categories, such as commonly used laboratory tests and imaging studies which may not be universally wasteful, but potentially overused.

While our prioritization framework focuses on cost and variability, another important factor when setting priorities is the reduction of unnecessary harm. Direct harm, such as side effects that result from medications, are easier to identify through traditional methods of study. However, indirect harms from unnecessary testing can also be significant but are generally more difficult to identify. The cascades of care have been described in other specialties in which unnecessary testing leads to further wasteful and harmful downstream care pathways [31–33]. In addition, invasive procedures have been associated with abnormalities on brain magnetic resonance imaging and lower IQ in preterm infants [34, 35]. While indirect harms from excess testing are less frequently discussed in neonatology, quality improvement efforts aimed at reducing unnecessary testing are in development and would directly address many of the top 10 CTT categories based on our prioritization score [36]. Future comparative effectiveness research and quality improvement efforts focused on optimizing testing patterns should consider the potential for indirect harm from excess testing.

Our study has several limitations. The PHIS database has a data quality control program to minimize the risk of data errors. We further minimized the risk of misclassification by applying exclusion criteria to improve data quality. However, using billing patterns to estimate variation in utilization may partly reflect differences in coding systems between hospitals. The PHIS database robustly combines different hospital billing definitions into a unified coding system and we further reduced miscoding risk by excluding extreme outlier hospitals, which may represent differences in billing practices that were unaccounted for. Cost-to-charge ratios are a commonly used tool to estimate cost, used by many large administrative databases like the National Inpatient Sample and the Kids Inpatient Database, and many economic evaluations [37–40]. Despite their prevalent use, they are not a precise estimate of cost [41]. The PHIS cost master index uses the mean of all hospital and department-specific estimated costs for each billable item to make dollar values directly comparable [15]. This adjustment should be considered when interpreting overall cost estimates. Our prioritization framework specifically and purposefully focused on potentially modifiable costs from clinician-ordered tests and treatments. Daily room costs have been shown to be the largest component of costs during birth hospitalization, and therefore the length of stay is a principal driver of cost [4]. Reducing the unnecessary length of stay is also critical to reducing costs and improving value in neonatal care.

There are also important limitations to the generalizability of these findings. The PHIS database is comprised of freestanding US children’s hospitals, which may not fully reflect practice patterns in other settings such as community birth hospitals where preterm infants often receive care. Higher-level neonatal units (commonly found within children’s hospitals) may spend more on patient care, even after adjusting for GA, outborn status, and patient mix [42]. This could bias our sample towards more costly care and may underestimate variability. Based on our specific inclusion criteria, our findings do not generalize to newborn populations with congenital anomalies, or to other NICU patient populations including late preterm and term infants. Similar analyses among those populations should be conducted to establish value-based improvement priorities for their care.

## Conclusion

We established a value-based prioritization framework for comparative effectiveness research and quality improvement based on cost, inter-hospital variability, and degree of exposure to different tests and treatment categories among VP and VLBW infants cared for in US children’s hospitals NICUs. We identified parenteral nutrition, anticoagulation, intravenous fluids, and frequently used laboratory and imaging modalities as top priorities for comparative effectiveness research and quality improvement efforts to increase the value of neonatal care.

## Supplementary information

Supplemental Index and Tables

Supplemental Figure 1

Supplemental Figure 2

## References

[1] Porter ME (2010). What is value in health care?. N Engl J Med.

[2] Dukhovny D, Pursley DM, Kirpalani HM, Horbar JH, Zupancic JAF (2016). Evidence, quality, and waste: solving the value equation in neonatology. Pediatrics.

[3] Ho T, Dukhovny D, Zupancic JAF, Goldmann DA, Horbar JD, Pursley DM (2015). Choosing wisely in newborn medicine: five opportunities to increase value. Pediatrics.

[4] King BC, Richardson T, Patel RM, Lee HC, Bamat NA, Patrick SW (2020). Cost of clinician-driven tests and treatments in very low birth weight and/or very preterm infants. J Perinatol.

[5] Goodman DC (2009). Unwarranted variation in pediatric medical care. Pediatr Clin North Am.

[6] Wennberg JE (2002). Unwarranted variations in healthcare delivery: implications for academic medical centres. Br Med J.

[7] Slaughter JL, Stenger MR, Reagan PB, Jadcherla SR (2016). Neonatal histamine-2 receptor antagonist and proton pump inhibitor treatment at United States Children’s Hospitals. J Pediatr.

[8] Slaughter JL, Stenger MR, Reagan PB (2013). Variation in the use of diuretic therapy for infants with bronchopulmonary dysplasia. Pediatrics.

[9] Stenger MR, Slaughter JL, Kelleher K, Shepherd EG, Klebanoff MA, Reagan P (2012). Hospital variation in nitric oxide use for premature infants. Pediatrics.

[10] Goodman D, Little G, Harrison W, Moen A, Mowitz M, Ganduglia-Cazaban C. The Dartmouth Atlas of neonatal intensive care. The Dartmouth Institute for Health Policy and Clinical Practice; 2019. 36264871

[11] Goodman DC, Ganduglia-Cazaban C, Franzini L, Stukel TA, Wasserman JR, Murphy MA (2019). Neonatal intensive care variation in medicaid-insured newborns: a population-based study. J Pediatr.

[12] Adams M, Bassler D, Bucher HU, Roth-Kleiner M, Berger TM, Braun J (2018). Variability of very low birth weight infant outcome and practice in swiss and US neonatal units. Pediatrics.

[13] Alleman BW, Bell EF, Li L, Dagle JM, Smith PB, Ambalavanan N (2013). Individual and center-level factors affecting mortality among extremely low birth weight infants. Pediatrics.

[14] Pierrat V, Burguet A, Marchand-Martin L, Cambonie G, Coquelin A, Roze JC, et al. Variations in patterns of care across neonatal units and their associations with outcomes in very preterm infants: the French EPIPAGE-2 cohort study. BMJ Open. 2020. 10.1136/bmjopen-2019-035075. 10.1136/bmjopen-2019-035075 PMC7311036 32571857

[15] Balakrishnan M, Raghavan A, Suresh GK (2017). Eliminating undesirable variation in neonatal practice. Clin Perinatol.

[16] Ho T, Zupancic JAF, Pursley DWM, Dukhovny D (2017). Improving value in neonatal intensive care. Clin Perinatol.

[17] Medicine I. Initial national priorities for comparative effectiveness research. The National Academies Press; 2009.

[18] Keren R, Luan X, Localio R, Hall M, McLeod L, Dai D (2012). Prioritization of comparative effectiveness research topics in hospital pediatrics. Arch Pediatr Adolesc Med.

[19] Cameron DB, Graham DA, Milliren CE, Glass CC, Feng C, Sidhwa F (2017). Quantifying the burden of interhospital cost variation in pediatric surgery. JAMA Pediatr.

[20] King BC, Richardson T, Patel RM, Lee HC, Bamat NA, Patrick SW, et al. Cost of clinician-driven tests and treatments in very low birth weight and/or very preterm infants. J. Perinatol. 2020. 10.1038/s41372-020-00879-6 33268831

[21] Dunn A, Grosse SD, Zuvekas SH (2018). Adjusting health expenditures for inflation: a review of measures for health services research in the United States. Health Serv Res.

[22] Richardson T, Rodean J, Harris M, Berry J, Gay JC, Hall M (2018). Development of hospitalization resource intensity scores for kids (H-RISK) and comparison across pediatric populations. J Hosp Med.

[23] Horbar JD, Edwards EM, Greenberg LT, Profit J, Draper D, Helkey D (2019). Racial segregation and inequality in the neonatal intensive care unit for very low-birth-weight and very preterm infants. JAMA Pediatr.

[24] Lee VS, Kawamoto K, Hess R, Park C, Young J, Hunter C (2016). Implementation of a value-driven outcomes program to identify high variability in clinical costs and outcomes and association with reduced cost and improved quality. J Am Med Assoc.

[25] Stowell C, Robicsek A. Endless forms most beautiful: evolving toward higher-value care. N Engl J Med Catal. 2018.

[26] Bonafide CP, Keren R (2018). Negative studies and the science of deimplementation. JAMA Pediatr.

[27] Wolf ER, Krist AH, Schroeder AR (2020). Deimplementation in pediatrics: past, present and future. JAMA Pediatr.

[28] Meakin G. Fluids exclusively enteral from day one in premature infants. ISRCTN Registry. 2019. http://www.isrctn.com/ISRCTN89654042.

[29] Bozkurt O, Alyamac Dizdar E, Bidev D, Sari FN, Uras N, Oguz SS. Prolonged minimal enteral nutrition versus early feeding advancements in preterm infants with birth weight ≤1250 g: a prospective randomized trial. J Matern Neonatal Med. 2020. 10.1080/14767058.2020.1716723 31994953

[30] Dorling J, Abbott J, Berrington J, Bosiak B, Bowler U, Boyle E (2019). Controlled trial of two incremental milk-feeding rates in preterm infants. N Engl J Med.

[31] Ganguli I, Lupo C, Mainor AJ, Raymond S, Wang Q, Orav EJ (2019). Prevalence and cost of care cascades after low-value preoperative electrocardiogram for cataract surgery in fee-for-service medicare beneficiaries. JAMA Intern Med.

[32] Ganguli I, Simpkin AL, Lupo C, Weissman A, Mainor AJ, Orav EJ (2019). Cascades of care after incidental findings in a US national survey of physicians. JAMA Netw Open.

[33] Grieme CV, Voss DR, Olson KE, Davis SR, Kulhavy J, Krasowski MD (2016). Prevalence and clinical utility of ‘incidental’ critical values resulting from critical care laboratory testing. Lab Med.

[34] Duerden E, Grunau R, Chau V, Groenendaal F, Guo T, Al E. Association of early skin breaks and neonatal thalamic maturation: a modifiable risk? Neurology. 2020. 10.1212/WNL.0000000000010953 PMC7836658 33087497

[35] Vinall J, Miller SP, Bjornson BH, Fitzpatrick KPV, Poskitt KJ, Brant R (2014). Invasive procedures in preterm children: Brain and cognitive development at school age. Pediatrics.

[36] Profit J, Scheid A, Ridout E. First do no harm: value-driven patient safety in the neonatal intensive care unit. Patient Saf Netw. 2019. https://psnet.ahrq.gov/perspective/first-do-no-harm-value-driven-patient-safety-neonatal-intensive-care-unit.

[37] Hay S, Mowitz M, Dukhovny D, Viner C, Levin J, King B, et al. Unbiasing costs? An appraisal of economic assessment alongside randomized trials in neonatology. Semin Perinatol. 2021. 10.1016/j.semperi.2021.151391. 10.1016/j.semperi.2021.151391 33583609

[38] Phibbs CS, Schmitt SK, Cooper M, Gould JB, Lee HC, Profit J (2019). Birth hospitalization costs and days of care for mothers and neonates in California, 2009–2011. J Pediatr.

[39] Russell RB, Green NS, Steiner CA, Meikle S, Howse JL, Poschman K (2007). Cost of hospitalization for preterm and low birth weight infants in the United States. Pediatrics.

[40] Zupancic JAF, Hibbs AM, Palermo L, Truog WE, Cnaan A, Black DM (2009). Economic evaluation of inhaled nitric oxide in preterm infants undergoing mechanical ventilation. Pediatrics.

[41] Shwartz M, Young DW, Siegrist R. The ratio of costs to charges: how good a basis for estimating costs? Inquiry; 1995. 8567084

[42] Rogowski J (2003). Using economic information in a quality improvement collaborative. Pediatrics.

