# Supplementary material for: Prioritization framework for improving the value of care for very low birth weight and very preterm infants
Source: J Perinatol. 2021 Jun 1;41(10):2463–73. doi: 10.1038/s41372-021-01114-6 (PMC8514333; doi:10.1038/s41372-021-01114-6)
Supplement: Supplementary file 1 — Supplemental Index and Tables [file 41372_2021_1114_MOESM1_ESM.docx]

**Supplemental Tables and Figures**

Supplemental Table 1 – Multiple logistic regression analysis excluding NICU-SOI Score

Supplemental Table 2 – Multiple linear regression analysis for CTT-related costs

Supplemental Figure 1 – Flowsheet of inclusion and exclusion criteria for patients included in the final cohort

Supplemental Figure 2 – Flowsheet of methodology for calculating the Prioritization Score

| Patient Demographics | Adjusted Probability of a Resource-Intensive NICU Stay (95% CI)^a^ | Adjusted Odds Ratio (95% CI) | P-value |
| --- | --- | --- | --- |
| Sex, N (%) |  |  |  |
| Male | 0.035 (0.022,0.057) | 1.38 (1.25,1.53) | <0.001 |
| Female | 0.026 (0.016,0.042) | REF |  |
| Gestational age (wks), N (%) |  |  |  |
| 22 | 0.088 (0.048,0.158) | 20.73 (10.36,41.49) | <0.001 |
| 23-24 | 0.138 (0.088,0.211) | 34.38 (19.93,59.29) | <0.001 |
| 25-26 | 0.073 (0.045,0.115) | 16.76 (9.82,28.60) | <0.001 |
| 27-28 | 0.027 (0.017,0.044) | 5.98 (3.54,10.09) | <0.001 |
| 29-30 | 0.014 (0.009,0.024) | 3.14 (1.88,5.26) | <0.001 |
| 31 | 0.010 (0.006,0.018) | 2.18 (1.23,3.87) | 0.008 |
| >31 weeks | 0.005 (0.002,0.009) | REF |  |
| Not Reported | 0.035 (0.019,0.062) | 7.74 (4.22,14.20) | <0.001 |
| Birth weight (g), N (%) |  |  |  |
| 400 - 499 | 0.197 (0.125,0.295) | 37.25 (23.48,59.11) | <0.001 |
| 500 - 749 | 0.101 (0.065,0.152) | 17.02 (11.77,24.61) | <0.001 |
| 750 - 999 | 0.046 (0.029,0.071) | 7.28 (5.11,10.38) | <0.001 |
| 1000 - 1249 | 0.020 (0.012,0.031) | 3.07 (2.19,4.30) | <0.001 |
| 1250 - 1499 | 0.013 (0.008,0.022) | 2.06 (1.49,2.87) | <0.001 |
| > 1499 g | 0.007 (0.004,0.011) | REF |  |
| Not Reported | 0.012 (0.003,0.050) | 1.79 (0.42,7.62) | 0.431 |
| Race/Ethnicity, N (%) |  |  |  |
| Non-Hispanic White | 0.037 (0.023,0.059) | REF |  |
| Non-Hispanic Black | 0.030 (0.018,0.048) | 0.80 (0.70,0.92) | 0.001 |
| Hispanic | 0.034 (0.021,0.056) | 0.93 (0.77,1.12) | 0.431 |
| Asian | 0.025 (0.014,0.044) | 0.67 (0.47,0.95) | 0.024 |
| Other | 0.027 (0.016,0.044) | 0.73 (0.61,0.87) | <0.001 |
| Admission Source, N (%) |  |  |  |
| Inborn | 0.019 (0.011,0.031) | REF |  |
| Outborn | 0.036 (0.022,0.057) | 1.94 (1.60,2.35) | <0.001 |
| Other | 0.041 (0.023,0.072) | 2.22 (1.44,3.42) | <0.001 |
| Age at Admission (days), N (%) |  |  |  |
| 0 | 0.029 (0.018,0.046) | REF |  |
| 1 | 0.031 (0.019,0.052) | 1.09 (0.88,1.35) | 0.425 |
| Insurance Type, N (%) |  |  |  |
| Commercial | 0.043 (0.027,0.067) | REF |  |
| Government | 0.041 (0.026,0.064) | 0.94 (0.84,1.06) | 0.323 |
| Self-pay | 0.016 (0.008,0.034) | 0.36 (0.20,0.67) | 0.001 |
| Other | 0.029 (0.015,0.053) | 0.66 (0.42,1.02) | 0.063 |
| Disposition at Discharge, N (%) |  |  |  |
| Home | 0.046 (0.029,0.073) | REF |  |
| Transfer | 0.056 (0.035,0.088) | 1.22 (1.05,1.41) | 0.009 |
| Mortality | 0.007 (0.004,0.012) | 0.15 (0.12,0.18) | <0.001 |
| Other/Unknown | 0.042 (0.021,0.085) | 0.91 (0.51,1.60) | 0.733 |
| Median Household Income Quartile |  |  |  |
| Q1 | 0.032 (0.020,0.052) | 1.04 (0.88,1.23) | 0.614 |
| Q2 | 0.029 (0.018,0.047) | 0.94 (0.80,1.12) | 0.502 |
| Q3 | 0.028 (0.017,0.046) | 0.92 (0.78,1.08) | 0.301 |
| Q4 | 0.031 (0.019,0.050) | REF |  |

**Supplemental Table 1** – Multiple Logistic Regression Analysis excluding NICU-SOI Score

| Patient Demographics | Adjusted Total CTT-related Cost (95% CI) | Adjusted Rate Ratio (95% CI) | P-value |
| --- | --- | --- | --- |
| Sex, N (%) |  |  |  |
| Male | $7 735 (6 681,8 956) | 1.06 (1.04,1.08) | <0.001 |
| Female | $7 289 (6 295,8 440) | REF |  |
| Gestational age (wks), N (%) |  |  |  |
| 22 | $6 147 (5 080,7 437) | 1.27 (1.11,1.46) | 0.001 |
| 23-24 | $10 715 (9 221,12 452) | 2.21 (2.07,2.36) | <0.001 |
| 25-26 | $11 750 (10 132,13 625) | 2.43 (2.30,2.57) | <0.001 |
| 27-28 | $9 739 (8 405,11 285) | 2.01 (1.92,2.11) | <0.001 |
| 29-30 | $7 741 (6 682,8 969) | 1.60 (1.53,1.67) | <0.001 |
| 31 | $6 255 (5 392,7 257) | 1.29 (1.23,1.36) | <0.001 |
| >31 weeks | $4 840 (4 159,5 634) | REF |  |
| Not Reported | $5 722 (4 885,6 703) | 1.18 (1.10,1.27) | <0.001 |
| Birth weight (g), N (%) |  |  |  |
| 400 - 499 | $7 474 (6 313,8 848) | REF |  |
| 500 - 749 | $6 683 (5 756,7 759) | 0.89 (0.82,0.97) | 0.011 |
| 750 - 999 | $7 213 (6 226,8 358) | 0.97 (0.88,1.06) | 0.443 |
| 1000 - 1249 | $7 428 (6 414,8 602) | 0.99 (0.90,1.09) | 0.899 |
| 1250 - 1499 | $7 849 (6 777,9 091) | 1.05 (0.95,1.16) | 0.333 |
| > 1499 g | $7 709 (6 642,8 948) | 1.03 (0.93,1.14) | 0.559 |
| Not Reported | $8 313 (6 868,10 061) | 1.11 (0.95,1.30) | 0.186 |
| Race/Ethnicity, N (%) |  |  |  |
| Non-Hispanic White | $7 841 (6 772,9 078) | REF |  |
| Non-Hispanic Black | $7 534 (6 502,8 729) | 0.96 (0.94,0.99) | 0.003 |
| Hispanic | $7 663 (6 609,8 886) | 0.98 (0.95,1.01) | 0.178 |
| Asian | $7 284 (6 239,8 504) | 0.93 (0.88,0.98) | 0.008 |
| Other | $7 240 (6 245,8 393) | 0.92 (0.90,0.95) | <0.001 |
| Admission Source, N (%) |  |  |  |
| Inborn | $7 065 (6 092,8 194) | REF |  |
| Outborn | $7 459 (6 451,8 625) | 1.06 (1.02,1.10) | 0.005 |
| Other | $8 034 (6 837,9 441) | 1.14 (1.04,1.24) | 0.003 |
| Age at Admission (days) N (%) |  |  |  |
| 0 | $7 421 (6 419,8 579) | REF |  |
| 1 | $7 598 (6 534,8 837) | 1.02 (0.98,1.07) | 0.308 |
| Insurance Type, N (%) |  |  |  |
| Commercial | $7 938 (6 870,9 172) | REF |  |
| Government | $7 848 (6 795,9 064) | 0.99 (0.97,1.01) | 0.301 |
| Self-pay | $6 565 (5 542,7 777) | 0.83 (0.75,0.91) | <0.001 |
| Other | $7 774 (6 626,9 121) | 0.98 (0.91,1.05) | 0.575 |
| Disposition at Discharge, N (%) |  |  |  |
| Home | $9 574 (8 286,11 063) | REF |  |
| Transfer | $9 182 (7 935,10 624) | 0.96 (0.93,0.99) | 0.003 |
| Mortality | $3 015 (2 603,3 491) | 0.31 (0.30,0.33) | <0.001 |
| Other/Unknown | $11 998 (10 050,14 323) | 1.25 (1.13,1.39) | <0.001 |
| Median Household Income Quartile |  |  |  |
| Q1 | $7 606 (6 565,8 812) | 1.03 (1.00,1.06) | 0.089 |
| Q2 | $7 536 (6 505,8 730) | 1.02 (0.99,1.05) | 0.254 |
| Q3 | $7 490 (6 464,8 678) | 1.01 (0.98,1.04) | 0.448 |
| Q4 | $7 406 (6 390,8 585) | REF |  |
| NICU-SOI Score | N/A | 1.48 (1.46,1.50) | <0.001 |

**Supplemental Table 2** – Multiple Linear Regression Analysis for CTT-related cost
