# Supplementary material for: Prioritization framework for improving the value of care for very low birth weight and very preterm infants
Source: J Perinatol. 2021 Jun 1;41(10):2463–73. doi: 10.1038/s41372-021-01114-6 (PMC8514333; doi:10.1038/s41372-021-01114-6)
Supplement: Supplementary file 2 — Supplemental Figure 1 [file 41372_2021_1114_MOESM2_ESM.pdf]

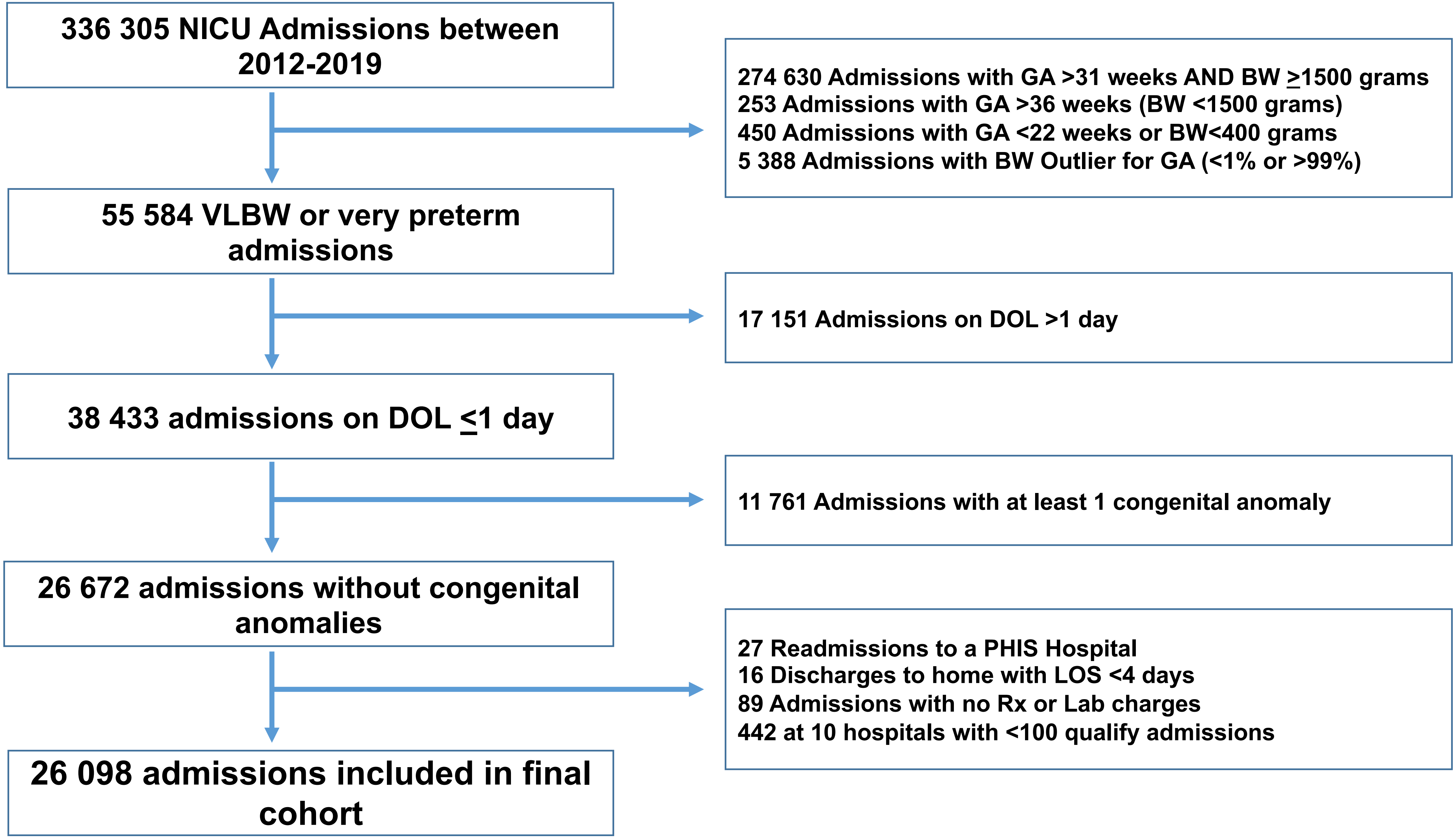

Supplemental Figure 1: Flowsheet of inclusion and exclusion criteria for patients included in the final cohort
