# Supplementary material for: Prioritization framework for improving the value of care for very low birth weight and very preterm infants
Source: J Perinatol. 2021 Jun 1;41(10):2463–73. doi: 10.1038/s41372-021-01114-6 (PMC8514333; doi:10.1038/s41372-021-01114-6)
Supplement: Supplementary file 3 — Supplemental Figure 2 [file 41372_2021_1114_MOESM3_ESM.pdf]

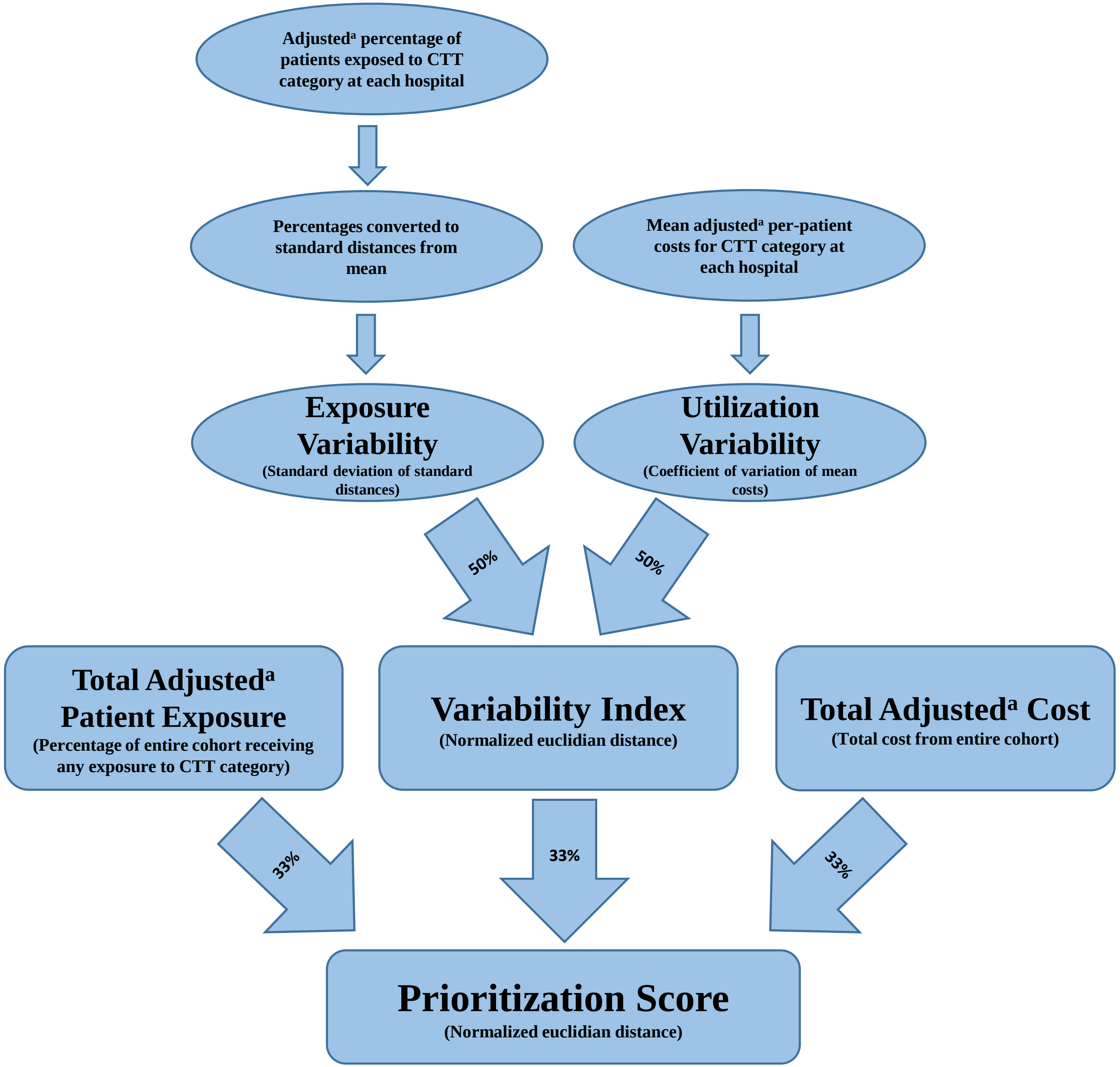

Supplemental Figure 2: Flowsheet of methodology for calculating the Prioritization Score

[a]Adjusted for patient demographics (GA, BW, Sex, Race/Ethnicity, Admission Source, Age at Admission, Median Household Income Quartile) and our NICU Severity of Illness Score
